# Left-ventricular volumes and ejection fraction from cardiac ECG-gated ^15^O-water positron emission tomography compared to cardiac magnetic resonance imaging using simultaneous hybrid PET/MR

**DOI:** 10.1007/s12350-022-03154-7

**Published:** 2022-12-08

**Authors:** Jonny Nordström, Sofia Kvernby, Tanja Kero, Jens Sörensen, Hendrik J. Harms, Mark Lubberink

**Affiliations:** 1 Department of Surgical Sciences/Nuclear Medicine & PET, Uppsala University, Uppsala, Sweden; 2 Centre for Research and Development, Uppsala/Gävleborg County, Gävle, Sweden; 3 Medical Imaging Centre, Uppsala University Hospital, Uppsala, Sweden; 4 Medical Physics, Uppsala University Hospital, Uppsala, Sweden

**Keywords:** LV volumes, ejection fraction, ^15^O-water, positron emission tomography, cardiac magnetic resonance imaging

## Abstract

**Background:**

^15^O-water PET is the gold standard for noninvasive quantification of myocardial blood flow. In addition to evaluation of ischemia, the assessment of cardiac function and remodeling is important in all cardiac diseases. However, since ^15^O-water is freely diffusible and standard uptake images show little contrast between the myocardium and blood pool, the assessment of left-ventricular (LV) volumes and ejection fraction (EF) is challenging. Therefore, the aim of the present study was to investigate the feasibility of calculating LV volumes and EF from first-pass analysis of ^15^O-water PET, by comparison with cardiac magnetic resonance imaging (CMR) using a hybrid PET/MR scanner.

**Methods:**

Twenty-four patients with known or suspected CAD underwent a simultaneous ECG-gated cardiac PET/MR scan. The ^15^O-water first-pass images (0-50 seconds) were analyzed using the CarPET software and the CMR images were analyzed using the software Segment, for LV volumes and EF calculations. The LV volumes and EF were compared using correlation and Bland–Altman analysis. In addition, inter- and intra-observer variability of LV volumes and EF were assessed for both modalities.

**Results:**

The correlation between PET and CMR was strong for volumes (*r* > 0.84) and moderate for EF (*r* = 0.52), where the moderate correlation for EF was partly due to the small range of EF values. Agreement was high for all parameters, with a slight overestimation of PET values for end-diastolic volume but with no significant mean bias for other parameters. Inter- and intra-observer agreement of volumes was high and comparable between PET and CMR. For EF, inter-observer agreement was higher for PET and intra-observer agreement was higher for CMR.

**Conclusion:**

LV volumes and EF can be calculated by first-pass analysis of a ^15^O-water PET scan with high accuracy and comparable precision as with CMR.

**Supplementary Information:**

The online version contains supplementary material available at 10.1007/s12350-022-03154-7.

## Introduction

Positron emission tomography (PET) is a beneficial tool in both diagnosis and prognosis of coronary artery disease (CAD) and is a recommended tool in the assessment of obstructive CAD and ischemia.^1,2^ Quantification of myocardial blood flow (MBF) has shown improved detection of significant CAD compared with qualitative analysis.^3–8^ In addition to evaluating ischemia, assessment of cardiac function and remodeling is important in all cardiac diseases, and evaluating cardiac volumes and function via ECG-gated PET images is now standard in most, if not all PET MBF procedures. Clinical representation of different remodeling patterns varies and the need for a classification is emphasized. For example, the end-diastolic volume (EDV) differentiates between concentric and eccentric remodeling patterns^9^ and the left-ventricular ejection fraction (LVEF) or LV systolic function is a powerful predictor of cardiac mortality on its own.^10–13^ Furthermore, differentiation of heart failure with reduced EF (HFrEF) from heart failure with preserved EF (HFpEF) is important since there exist clinically proven treatments to improve morbidity and mortality in HFrEF but for HFpEF there is only the recently approved SGLT2-inhibitors.^14,15^

For PET uptake tracers including ^82^Rb and ^13^N-ammonia, LV volumes and EF are routinely assessed through ECG-gated late-uptake images.^16,17^ This is not feasible for ^15^O-water, while considered the gold standard for noninvasive MBF measurements, the lack of net uptake rules out the use of such ECG-gated late-uptake images. The feasibility of calculating LV volumes and EF from ^15^O-water PET has been shown in two previous studies utilizing ECG-gated first-pass or parametric images.^18,19^ In one of those studies, the optimal time range for the first pass was defined after inspection of the dynamic images, resulting in ECG-gated images of the time points in which the ^15^O-water is confined to the left side of the heart. The other study used a time consuming approach and imported ECG-gated parametric or first-pass images into a commercially available SPECT software. While both studies showed promising results toward the feasibility of calculating LV volumes and EF, their approaches are less suitable for a rapid clinical workflow.

In this study, a faster approach ready for clinical implementation is presented by using a standardized fixed time range for the first pass and a highly automated analysis performed in the same software used for standard MBF calculations. Cardiac magnetic resonance imaging (CMR) is considered the gold standard for volume and ejection-fraction estimation due to the method’s excellent differentiation of myocardium from the blood pool and the lung tissue with high reproducibility of volume estimates. ^20–22^ In this study, LV volumes and EF were assessed with ^15^O-water PET and CMR simultaneously in a hybrid PET/MR scanner with the aim to verify a method for measurement of LV volumes and EF calculations from ^15^O-water PET feasible for clinical practice. To the best of our knowledge, this is the first study that assessed LV volumes and EF from ^15^O-water PET simultaneously with CMR using a hybrid PET/MR, which should be considered the ultimate reference standard.

## Methods

### Patients

Twenty-four patients with known or suspected CAD were included. All had intermediate pre-test probability of CAD according to ESC guidelines 2013^23^ and none had any known history of ST-elevation myocardial infarction. Patient characteristics are described in Table 1. Written, informed consent was obtained from all subjects and the study was performed with permission from the local Radiation Ethics Committee and the Regional Board of Medical Ethics in Uppsala, and in accordance with the Declaration of Helsinki.

**Table 1:** Patient characteristics

Age (mean ± SD)	64.3 ± 8.8 years
Sex	15 male, 9 female
Weight (mean ± SD)	82.4 ± 17.6 kg
Diastolic blood pressure (mean ± SD)	75.4 ± 10.2 mmHg
Systolic blood pressure (mean ± SD)	133.2 ± 16.2 mmHg
Hypertension (yes/no)	16/8
Hyperlipidemia (yes/no)	18/6
Diabetes (yes/no)	6/18
Smoking (yes/no)	3/21
Hypokinesia or akinesia (yes/no)	2/22

### Image acquisition

All patients underwent a simultaneous ECG-gated cardiac PET/MR scan. Data were acquired using an integrated 3 T PET/MR scanner (Signa PET/MR, GE Healthcare, Waukesha).

#### PET

A standard clinical protocol was used where a 6 minutes ECG-gated scan, acquired in list mode, was started simultaneously with an automated fast bolus injection of 400 MBq ^15^O-water (10 mL ^15^O-water at 0.8 mL⋅s followed by 30 mL saline at 2 mL⋅s). A two-point Dixon sequence was acquired during end-expiration breath hold for the creation of an attenuation correction map. Images were reconstructed using a time-of-flight ordered subsets expectation maximization (2 iterations, 28 subsets) algorithm both as gated first-pass images and as nongated dynamic images sorted into 22 frames (1 × 10 s, 8 × 5 s, 4 × 10 s, 2 × 15 s, 3 × 20 s, 2 × 30 s, and 2 × 60 s). Data from the first 10 to 50 seconds of the scan were reconstructed as 8-bin cardiac-gated blood-pool images.

#### CMR

A balanced steady-state gradient-echo (FIESTA) cine sequence was acquired in short-axis view during breath hold (TR: 3.155 ms, TE: 1.112 ms, flip angle: 50°, FOV_xy_: 380 mm, in plane pixel size: 1.48 mm, and slice thickness: 8 mm). The whole heart was covered in 10 to 12 slices, depending on the size of the heart. Data were ECG gated, and the cardiac cycle was divided into 20 gating bins.

### Volumes and EF calculations

#### PET

PET images were analyzed using a prototype first-pass gating module in the in-house CarPET software. First, the left and right side of the heart in the first-pass images were separated using a steepest-path approach.^24^ In this approach, all voxels follow the steepest path to a local maximum, and all voxels reaching the same maximum are considered a seed. Combining these seeds with parametric images of the arterial (*V*_A_) and venous (*V*_V_) spill-over fraction, computed in the MBF analysis,^25^ enables each seed to be assigned to the left or right heart, based on the *V*_A_ and *V*_V_ value of the voxel with the maximum of each seed, respectively.

**Figure 1:**
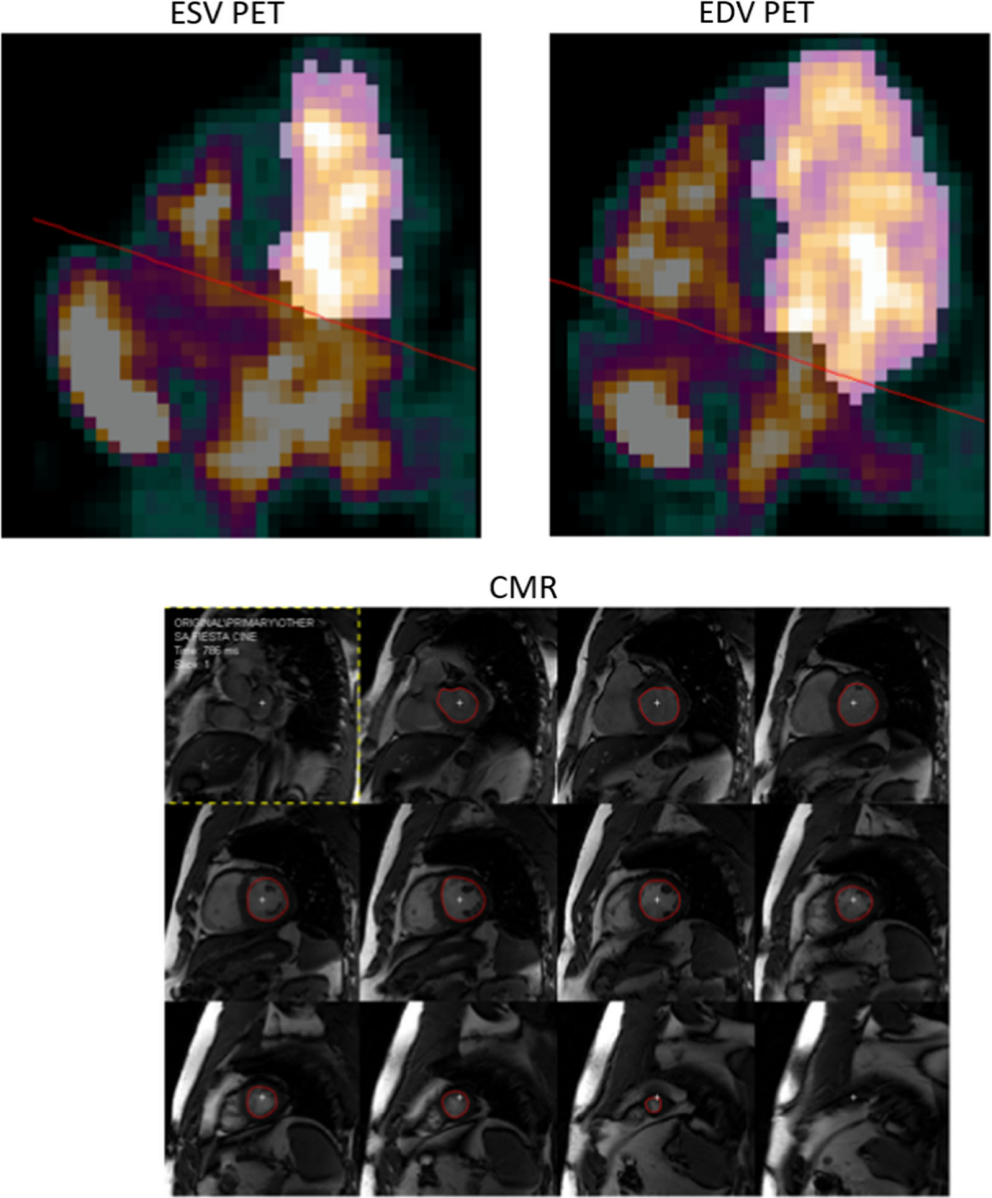
Example image showing the segmentation of one patient’s first-pass images from ^15^O-water PET (top row) and corresponding segmentation of the same patient’s CMR (bottom row). Highlighted regions of the PET images are voxels included in each volume

It is assumed that the cardiac valves, while below the resolution of the PET scanner, result in a lowered ^15^O-water concentration, and voxels on either side of the valves will reach a different seed. Identifying seeds on either side of the mitral valve plane will result in identification of the LV cavity. To identify the likely location of the valve plane, indentations in the long-axis profile are identified for multiple angles around the long axis. For each angle, a mask of low-activity voxels is defined and the distance from one to the other side is computed. Local minima in this distance are considered an indentation in the activity, and at one of these indentations is assumed to be the atrioventricular border. Once a set of possible indentations are found, the set most likely to represent the border is identified and a 3D plane is fitted through the points. This 3D plane is stored as initial suggestion of the mitral valve plane, which the user can adjust prior to final calculations of the cardiac volumes. In Fig. 1S, a graphical representation is shown.

Finally, all seeds on the apical side of the valve plane were included in the final volume, and final volumes of EDV and end-systolic volume (ESV) were obtained using a count-based approach. Stroke volume (SV) was calculated as SV = EDV − ESV and ejection fraction as EF = SV/EDV.

The analysis was performed blinded by two observers to estimate the inter-observer variability of the method. For estimation of the intra-observer variability, one of the observers performed a second blinded analysis of the data. To avoid recognition bias, a time interval of at least 4 weeks passed between the first and second intra-observer analyses.

#### CMR

CMR images were analyzed using the freely available software Segment (Medviso AB, Lund).^26^ Endocardial borders were manually contoured in short-axis view, both in the end-diastolic and end-systolic phase of the cardiac cycle. The trabeculae and papillary muscles volume were included in the left-ventricular blood pool. The most basal slice needed to display at least 50% visible myocardial circumference to be included in the analysis. The LV outflow tract was included in the blood volume by using the aortic valve as the lateral border when drawing the endocardial contour. Stroke volume was calculated as SV = EDV − ESV and ejection fraction as EF = SV/EDV.

LV contouring was performed blinded by two observers to estimate the inter-observer variability of the method. For estimation of the intra-observer variability, one of the observers performed a second blinded analysis of the data. To avoid recognition bias, a time interval of at least 4 weeks passed between the first and second intra-observer analyses.

### Statistics

Data are presented as mean values ± standard deviation (SD). Correlation of LV volumes and EF from PET compared with CMR was assessed using linear regression and the Pearson’s correlation coefficient (*r*). Agreement was assessed using Bland–Altman analysis, the nonparametric paired Wilcoxon sign-rank test, and the intra-class correlation coefficient (ICC). The repeatability coefficient (RPC) was calculated as 1.96 × SD of differences. Inter- and intra-observer variability was assessed using ICC for agreement and Bland–Altman analysis. Statistical analysis was performed in Matlab (The Mathworks, Natick, Massachusetts).

## Results

**Figure 2:**
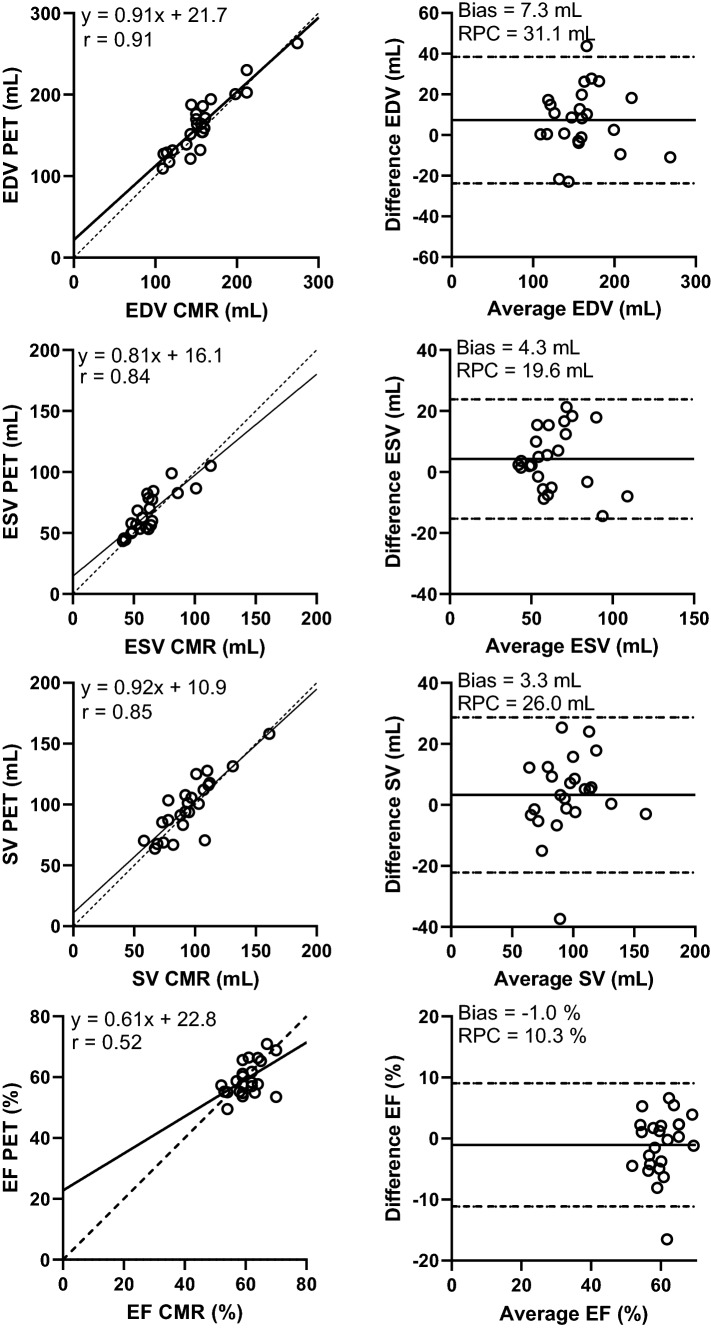
Linear-regression and Bland–Altman analysis between PET and CMR for all parameters

**Figure 3:**
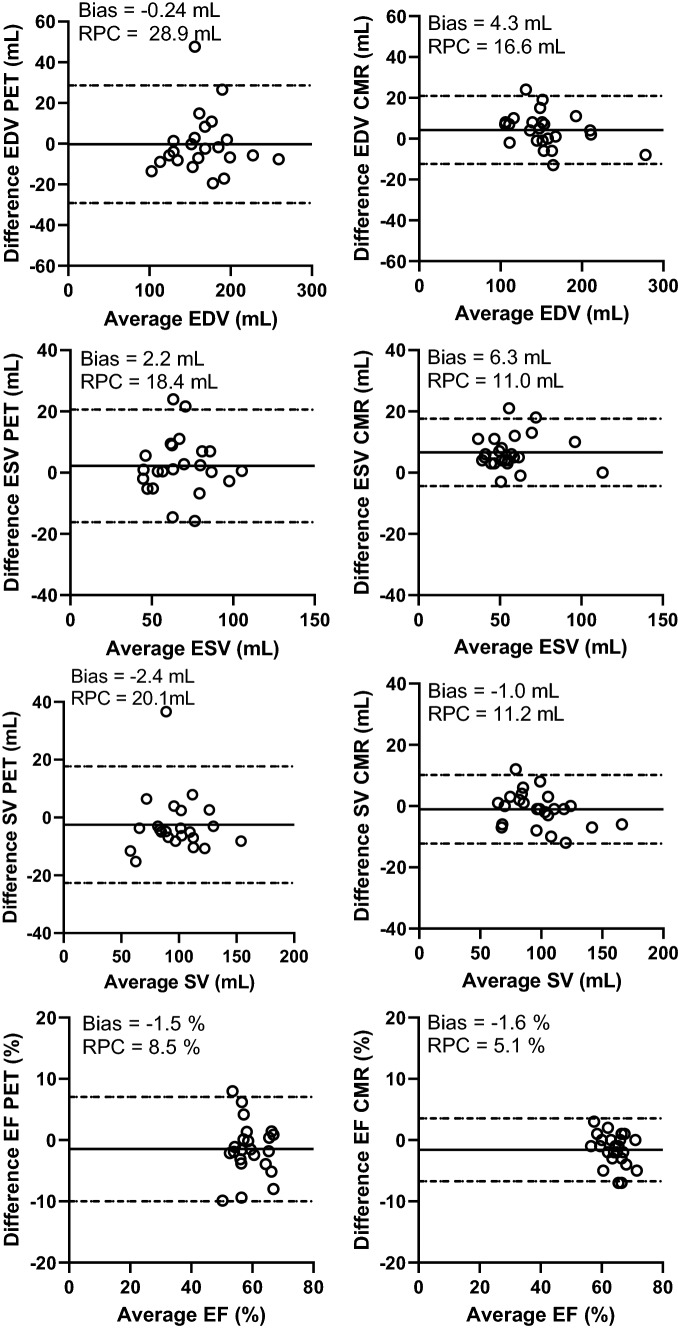
Bland–Altman analysis showing inter-observer variability for PET (left panel) and CMR (right panel)

Figure 1 shows example images of the segmentation of one patient’s PET and CMR. The range of measured LV volumes and EF were for CMR: 109 to 274 mL (EDV), 41 to 113 mL (ESV), and 52 to 70% (EF). For PET, the range of measured LV volumes and EF values were: 109 to 263 mL (EDV), 43 to 105 mL (ESV), and 49 to 71% (EF). One patient’s data had to be excluded from the results due to technical issues with the gated image analysis for PET. The gated analysis of PET was time efficient with no more than a few minutes analysis time per patient. Correlations between PET and CMR were strong for EDV, ESV, and SV, and moderate for EF (Fig. 2; Table 2). On average, EDV from PET data was slightly overestimated but ESV, SV, and EF showed no significant bias. ICC between modalities was moderate for EF, good for ESV and SV, and excellent for EDV. Figure 3 presents Bland–Altman plots of inter-observer variability for PET and CMR and Table 3 presents ICC values for inter- and intra-observer variability. For PET, inter- and intra-observer agreement was moderate for EF (ICC = 0.68) and good to excellent for EDV, ESV and SV (ICC > 0.85). Inter- and intra-observer agreement of volumes was high and comparable between PET and CMR. For EF, inter-observer agreement was higher for PET and intra-observer agreement was higher for CMR.

**Table 2:** Relative mean bias, ICC, and *r*^2^ between PET and CMR for all measured parameters

	Average PET	Average CMR	Bias (%)	ICC	*r*
EDV	164 ± 37 mL	156 ± 37 mL	5.2 ± 10.5*	0.90	0.91
ESV	66 ± 17 mL	61 ± 18 mL	8.3 ± 15.5	0.82	0.84
SV	98 ± 24 mL	95 ± 22 mL	4.0 ± 14.1	0.84	0.85
EF	60 ± 6%	61 ± 5%	− 1.5 ± 8.1	0.51	0.52

*EDV*, end-diastolic volume; *ESV*, end-systolic volume; *SV*, stroke volume; *EF*, ejection fraction; *ICC*, intra-class correlation coefficient

*P < .05

**Table 3:** Intraclass correlation coefficient (ICC) for inter- and intra-observer variability for PET respectively CMR

	Inter-observer PET	Intra-observer PET	Inter-observer CMR	Intra-observer CMR
EDV	0.93 (0.83–0.97)	0.94 (0.86–0.97)	0.97 (0.92–0.99)	0.99 (0.97–1.0)
ESV	0.85 (0.69–0.94)	0.93 (0.85–0.97)	0.89 (0.25–0.97)	0.96 (0.70–0.99)
SV	0.91 (0.79–0.96)	0.86 (0.70–0.94)	0.93( 0.84–0.97)	0.98 (0.95–0.99)
EF	0.68 (0.39–0.85)	0.68 (0.68–0.85)	0.50 (0.04–0.77)	0.82 (0.40–0.94)

*EDV*, end-diastolic volume; *ESV*, end-systolic volume; *SV*, stroke volume; *EF*, ejection fraction

There is one outlier in the results where PET clearly underestimates EF and SV. For this patient, the results showed high inter-observer agreement for both PET and CMR. No explanation for the underestimation of values by PET could be found. If the outlier was removed from the results correlation and/or agreement increased for SV (*r* = 0.85 to *r* = 0.91, ICC = 0.84 to ICC = 0.89) and EF (*r* = 0.52 to *r* = 0.70, ICC = 0.51 to ICC = 0.69).

## Discussion

^15^O-water PET is the gold standard for noninvasive quantification of MBF, but implementation of simple, routine quantification of cardiac function via EF has not been feasible. In this study, we show the feasibility of calculating LV volumes and EF from ^15^O-water PET by comparison with CMR, using an integrated hybrid PET/MR scanner, and without the need for scan-specific reconstruction windows. Instead, a standardized time window from 10 to 50 seconds was used for every patient, which had been optimized to include a high uptake in the right- and left-ventricular cavities but with limited uptake in the myocardium.

In this study, correlation between ^15^O-water PET and CMR was strong for LV volumes but clearly weaker for EF. This was not surprising since the range of EF values was small. This study only included patients with an intermediate likelihood of CAD, no known myocardial infarctions, and was therefore limited to patients with normal systolic function. Agreement between ^15^O-water PET and CMR was high for all parameters with a small but significant overestimation of ^15^O-water PET values for EDV. The RPC was in the same range as inter-observer variability for all parameters but lower than in previous studies comparing ^15^O-water PET to CMR.^18,19^ As the images were obtained simultaneously, physiological variability between PET and CMR can be considered negligible and only technical variability remains, which likely explains the improved RPC in this study.

Assessment of LV volumes and EF in nuclear cardiology is typically based on segmentation of the myocardial wall using cardiac-gated images of tracer retention, a methodology that is conceptually similar to the CMR approach. Utilizing this method, gated SPECT has been used for a long time in clinical routine and has shown good agreement with CMR.^27,28^ In this study, agreement in terms of mean bias and RPC of ^15^O-water PET was similar or superior to the results of the previous studies comparing SPECT to CMR. Comparing PET to CMR using cardiac-gated late-uptake images of ^13^N-ammonia, two recent studies showed agreement of LV volumes in the same range or lower than the results of the present study.^29,30^ Segmentation of images showing myocardial retention is challenged in patients with severe perfusion defects inducing errors in LV volume and EF calculations.^31^ Blood pool based methods, like the current one, have the benefit of eliminating this error.

Assessment of LV volumes and EF with CMR have in other studies demonstrated an excellent reproducibility.^22^ LV volumes and EF assessment from ^15^O-water PET in this study, provided a reproducibility in the same range as CMR when evaluated as inter-observer variability, though intra-observer variability was slightly higher for CMR than for ^15^O-water PET. As the ^15^O-water technique is new, the observers had no experience in the PET analysis. The current ^15^O-water PET method is highly automated and the only manual adjustment needed is fine tuning of the atrioventricular plane location. In some cases, this poses a challenge when an additional seeding cluster close to the plane location is either included or excluded in the blood-pool volume after a small adjustment of the plane location, and therefore introduces variability in the measurement. Alternative or optimized segmentation algorithms may further improve the observer variability.

Even if agreement of LV volumes and EF between ^15^O-water PET and CMR was high, there were differences in the results between PET and CMR, displayed by the Bland–Altman plots and RPC. The differences might be due to several reasons. The number of cardiac gates differ between the methods, with 8 gates for PET and 20 gates for CMR. This may affect the quality of imaging and how accurate the delineation of the myocardial wall can be performed. The fewer cardiac gates, the larger the uncertainty becomes in determining when end-diastole or end-systole occurs, which implies that the results of the volume estimation at these time points may be affected. When using only 8 gates, ESV is typically overestimated which is also seen in the result, though not statistically significant. Increasing the number of gates would be desirable but the resulting low count statistics in each gate eliminate that possibility at present. Increasing the amount of injected activity would improve count statistics but to the cost of increased radiation dose. However, the radiation dose of ^15^O-water is fairly low (~ 0.1 mSv per 100 MBq) so increasing the activity comes at a fairly low cost of additional radiation dose, and the trade-off between image quality versus the ALARA principle could be revisited. Furthermore, the spatial resolution differs between the methods where PET is limited by a low resolution compared to CMR, but on the other hand, PET has the advantage of being a very sensitive method where even small changes in tracer uptake can be identified and implemented in model calculation for improving the estimation of functional measures.^32^ Also, the inherent difference of blood-pool segmentation of the PET images to edge detection of the CMR images may contribute to an impaired agreement of the two methods. However, as CMR is the gold standard for LV volumes and EF estimates, it is the measure we want to compare to for a proper clinical validation. Finally, EDV was the only parameter with a statistically significant mean bias. The reason for this is unclear but it is probably inherent to the implementation of the method. The seed approach requires a gradient, which is present when the atrioventricular valves are closed. EDV is the time point at which the valves close and if a bin is chosen when the valves are still open, it may challenge the valve plane positioning.

The PET analysis method used in this study is highly automated and can be added as an additional analysis to the standard MBF quantification with a small increase of analysis time. The gated analysis takes no more than a few minutes which is in the same range or shorter than a typical CMR analysis. It could thereby easily be implemented in the clinical workflow of ^15^O-water PET evaluations. For evaluation of concomitant structural heart disease such as LV dilation or decreased systolic function, echocardiography or CMR are recommended as the baseline test in recent guidelines.^1^ However, there are several clinical scenarios were assessment of LV volumes and EF from PET would add clinical value, e.g., for patients with inconclusive echocardiography and contraindications for CMR, when PET is performed prior to echocardiography, or when PET is performed long after echocardiography.

Limitations of the present study should be noted. The standardized time window of 10 to 50 seconds is beneficial in terms of automatization, decreased analysis time, and clinical utilization, but it does include one drawback. With a fixed time window, contrast between the right- and left ventricle and the myocardium is not individually optimized for every patient. For that, a scan-specific time window would need to be manually determined for every patient, which would impair the automatization and clinical utility of the present method. However, the fixed time window did work for every patient. Further on, the cohort is limited to patients with suspected or know CAD with preserved ejection fraction and further evaluations in other cohorts and in patients with reduced systolic function is needed. Nevertheless, it is not expected that larger hearts and/or poor systolic function would challenge the segmentation algorithm, as long as the atrioventricular border can be identified. The only challenge could be the definition of the timing interval of the first pass. For patients with known poor cardiac function, a longer interval may be used as the reduction in cardiac function and cardiac output may result in longer cardiopulmonary transit times and a later arrival of the bolus in the LV. For patients with EF on the high end of the range (i.e., small systolic volumes), partial volume effects may play a role, reducing in smaller physical volumes. However, as the software uses a low threshold count-based approach, the calculated volumes should still be accurate.

Moreover, estimation of LV volumes and EF were performed in patients only during rest. Assessment of EDV and EF during stress could add information on transient ischemic dilation or a reduced EF, both of which are markers of advanced cardiac disease. However, stress induces challenges with increased atrioventricular plane motion as well as patient motion, and the time window for reconstruction of the first pass cannot be standardized as for rest. Therefore, further evaluation of the current methods feasibility during stress is needed.

## Conclusion

LV volumes and EF can be calculated from a single ^15^O-water PET scan with high accuracy and comparable precision as with CMR. The method is fast, highly automated, requires minimal user intervention and could be implemented as a part of the clinical standard analysis of ^15^O-water PET.

## New knowledge gained

LV volumes and EF can be calculated from ^15^O-water PET utilizing an approach that is automated and fast enough for implementation in clinical practice.

## Supplementary Information

Below is the link to the electronic supplementary material.

Supplementary file1 (DOCX 132 kb)

Supplementary file2 (PPTX 346 kb)

Supplementary file3 (MP3 7139 kb)

## Abbreviations

MBF: Myocardial blood flow
PTF: Perfusable tissue fraction
LV: Left ventricle
EDV: End-diastolic volume
ESV: End-systolic volume
SV: Stroke volume
EF: Ejection fraction
PET: Positron emission tomography
CMR: Cardiac magnetic resonance
ICC: Intraclass correlation coefficient
RPC: Repeatability coefficient
