# Supplementary material for: Left-ventricular volumes and ejection fraction from cardiac ECG-gated 15O-water positron emission tomography compared to cardiac magnetic resonance imaging using simultaneous hybrid PET/MR
Source: J Nucl Cardiol. 2022 Dec 8;30(4):1352–62. doi: 10.1007/s12350-022-03154-7 (PMC10372106; doi:10.1007/s12350-022-03154-7)
Supplement: Supplementary file 1 — Supplementary file1 (DOCX 132 kb) [file 12350_2022_3154_MOESM1_ESM.docx]

**
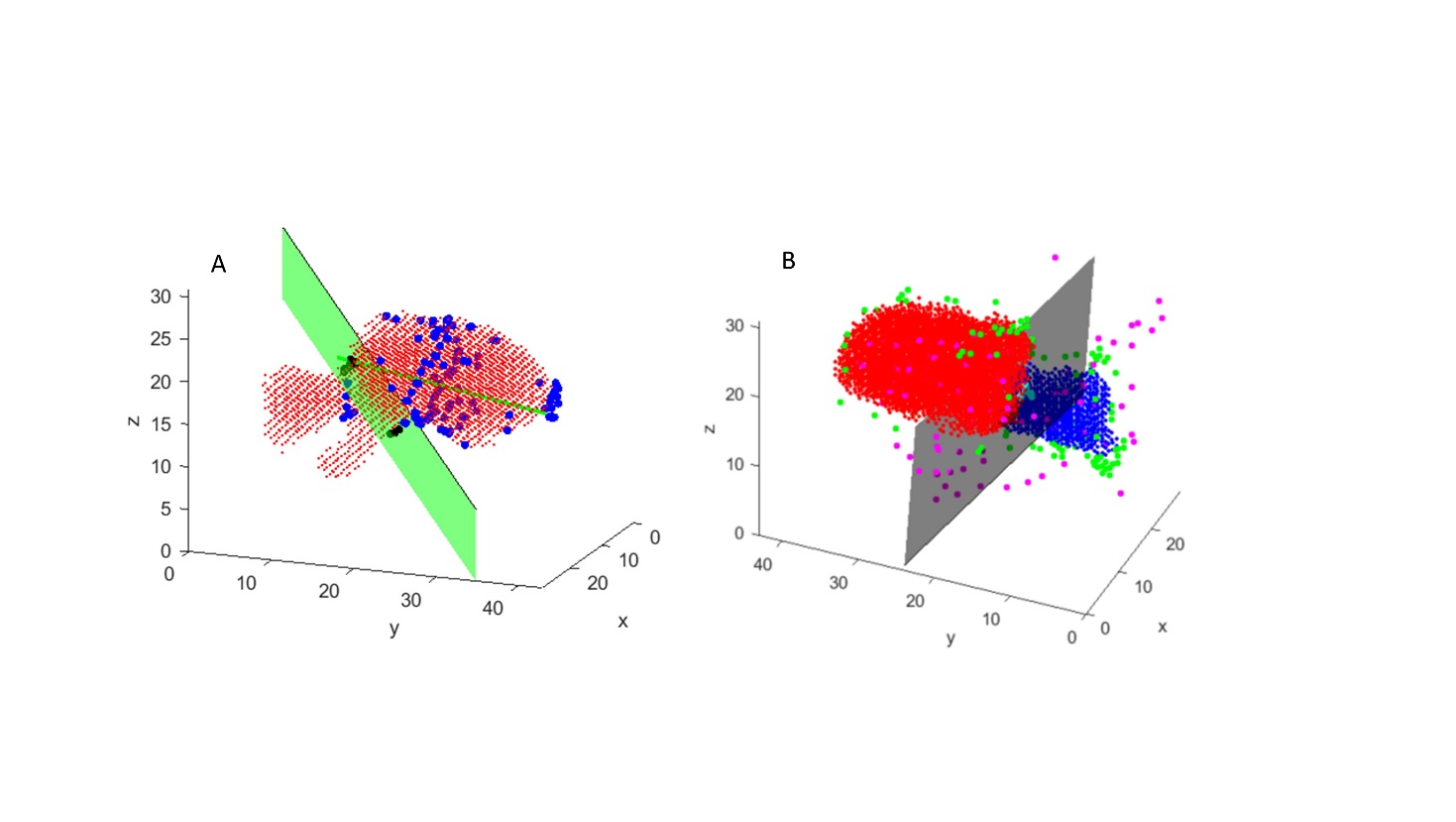
**Figure 1S. Graphical representation of the mitral valve plane positioning of the software. In A, the blue points show indentations of the LV blood pool that are detected by the software, concentrated along the apex, the papillary muscles and the valve plane. These are all obtained by taking 2D projections around the long axis (green line) at various angles. The green 3D plane is fitted through the points identified to be located at the mitral valve plane position, defined as the group of points closest to the aorta. In B, the pink and green points are seed points. Those located outside of the cavities are points below the threshold and are not segmented. All seeds whose seed points are on the LV side of the valve plane are included in the counts based algorithm and are displayed in red. All other seeds are displayed in blue.
