# Supplementary material for: Left-ventricular volumes and ejection fraction from cardiac ECG-gated 15O-water positron emission tomography compared to cardiac magnetic resonance imaging using simultaneous hybrid PET/MR
Source: J Nucl Cardiol. 2022 Dec 8;30(4):1352–62. doi: 10.1007/s12350-022-03154-7 (PMC10372106; doi:10.1007/s12350-022-03154-7)
Supplement: Supplementary file 2 — Supplementary file2 (PPTX 346 kb) [file 12350_2022_3154_MOESM2_ESM.pptx]

## Slide 1
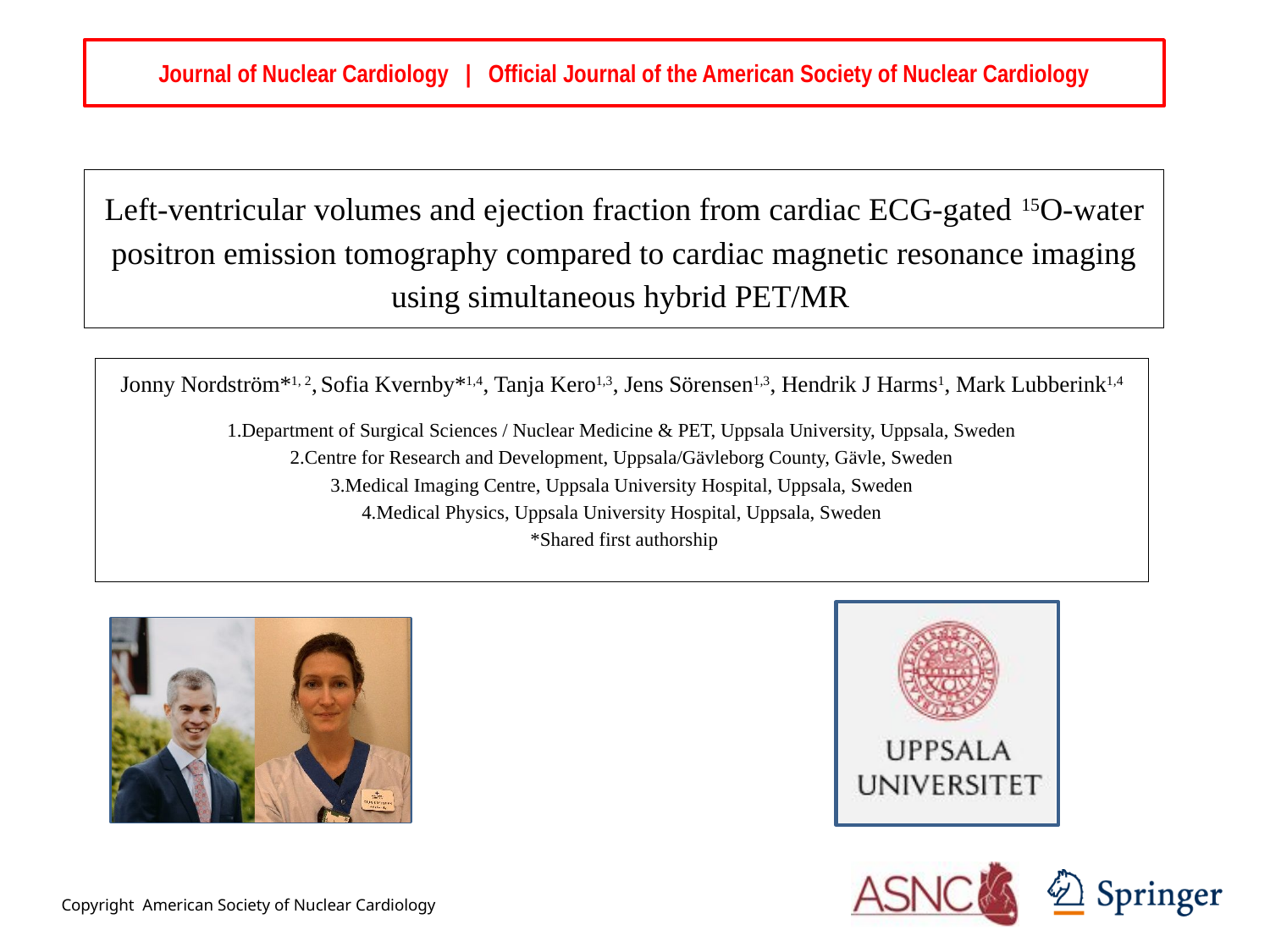

Journal of Nuclear Cardiology | Official Journal of the American Society of Nuclear Cardiology
# Left-ventricular volumes and ejection fraction from cardiac ECG-gated 15O-water positron emission tomography compared to cardiac magnetic resonance imaging using simultaneous hybrid PET/MR
Jonny Nordström*1, 2, Sofia Kvernby*1,4, Tanja Kero1,3, Jens Sörensen1,3, Hendrik J Harms1, Mark Lubberink1,4
1.Department of Surgical Sciences / Nuclear Medicine & PET, Uppsala University, Uppsala, Sweden
2.Centre for Research and Development, Uppsala/Gävleborg County, Gävle, Sweden
3.Medical Imaging Centre, Uppsala University Hospital, Uppsala, Sweden
4.Medical Physics, Uppsala University Hospital, Uppsala, Sweden
 *Shared first authorship
Copyright American Society of Nuclear Cardiology

## Slide 2
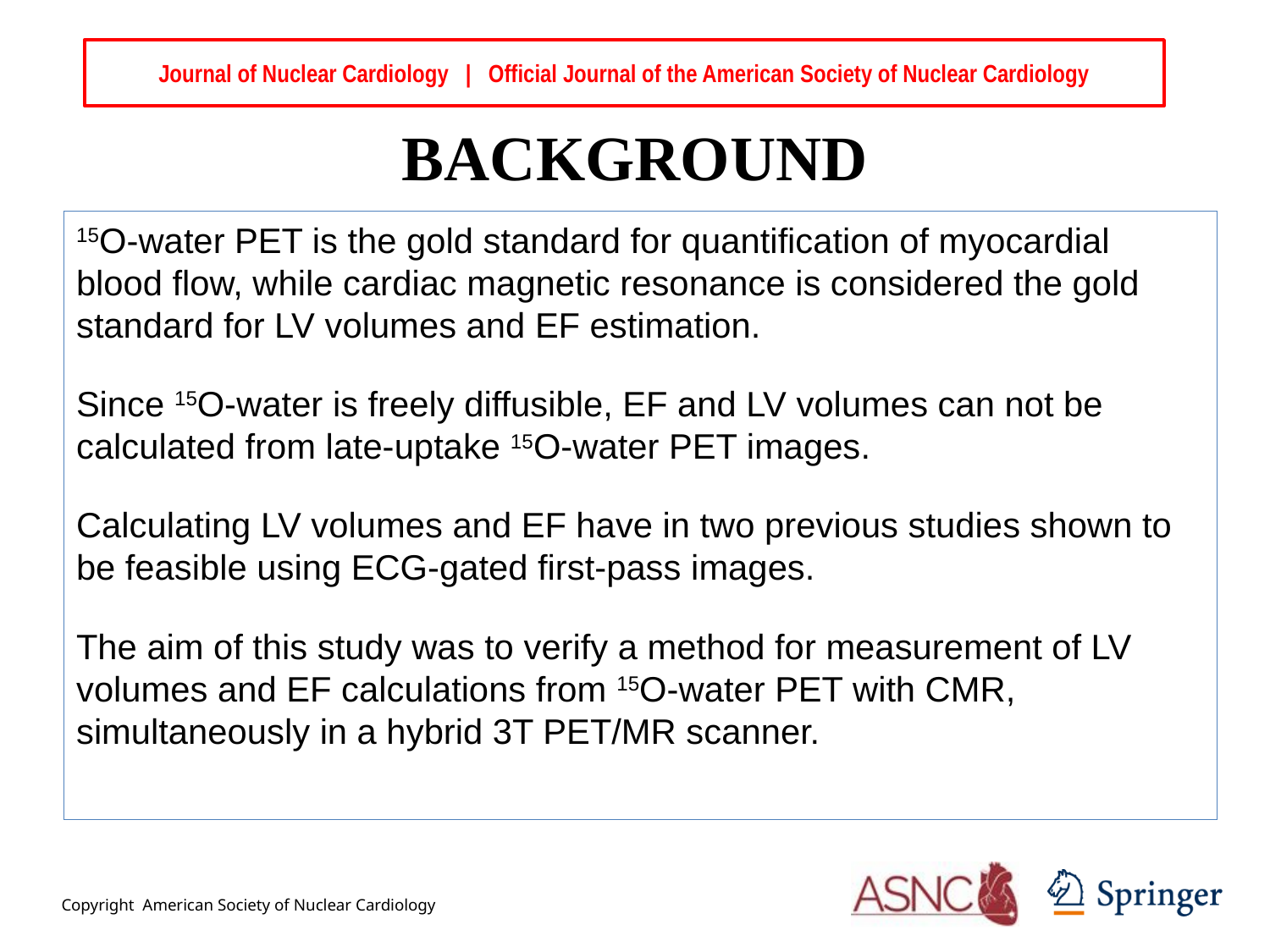

Journal of Nuclear Cardiology | Official Journal of the American Society of Nuclear Cardiology
# BACKGROUND
15O-water PET is the gold standard for quantification of myocardial blood flow, while cardiac magnetic resonance is considered the gold standard for LV volumes and EF estimation.
Since 15O-water is freely diffusible, EF and LV volumes can not be calculated from late-uptake 15O-water PET images.
Calculating LV volumes and EF have in two previous studies shown to be feasible using ECG-gated first-pass images.
The aim of this study was to verify a method for measurement of LV volumes and EF calculations from 15O-water PET with CMR, simultaneously in a hybrid 3T PET/MR scanner.
Copyright American Society of Nuclear Cardiology

## Slide 3
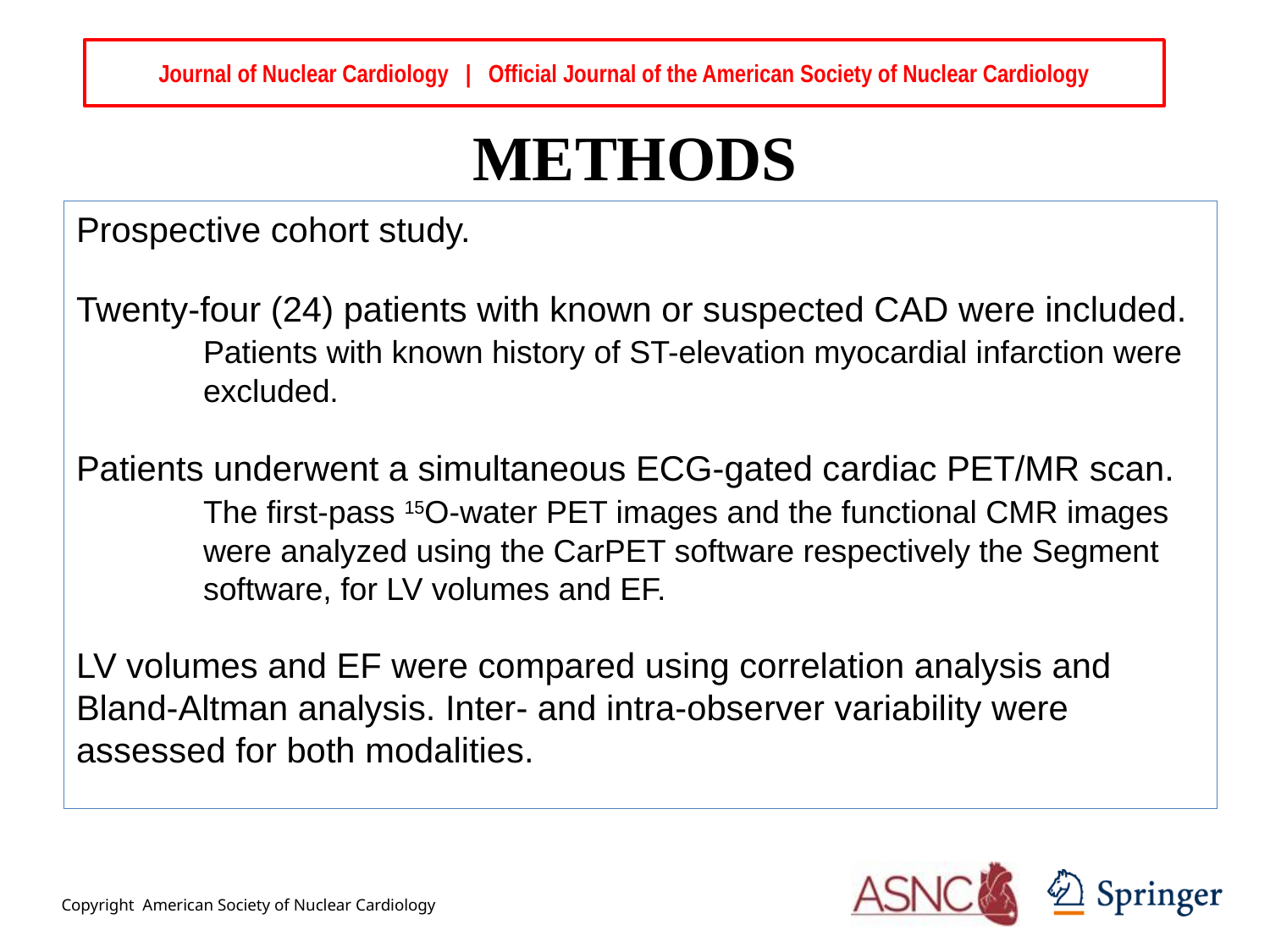

Journal of Nuclear Cardiology | Official Journal of the American Society of Nuclear Cardiology
# METHODS
Prospective cohort study.
Twenty-four (24) patients with known or suspected CAD were included. 	Patients with known history of ST-elevation myocardial infarction were 	excluded.
Patients underwent a simultaneous ECG-gated cardiac PET/MR scan. 	The first-pass 15O-water PET images and the functional CMR images 	were analyzed using the CarPET software respectively the Segment 	software, for LV volumes and EF.
LV volumes and EF were compared using correlation analysis and Bland-Altman analysis. Inter- and intra-observer variability were assessed for both modalities.
Copyright American Society of Nuclear Cardiology

## Slide 4
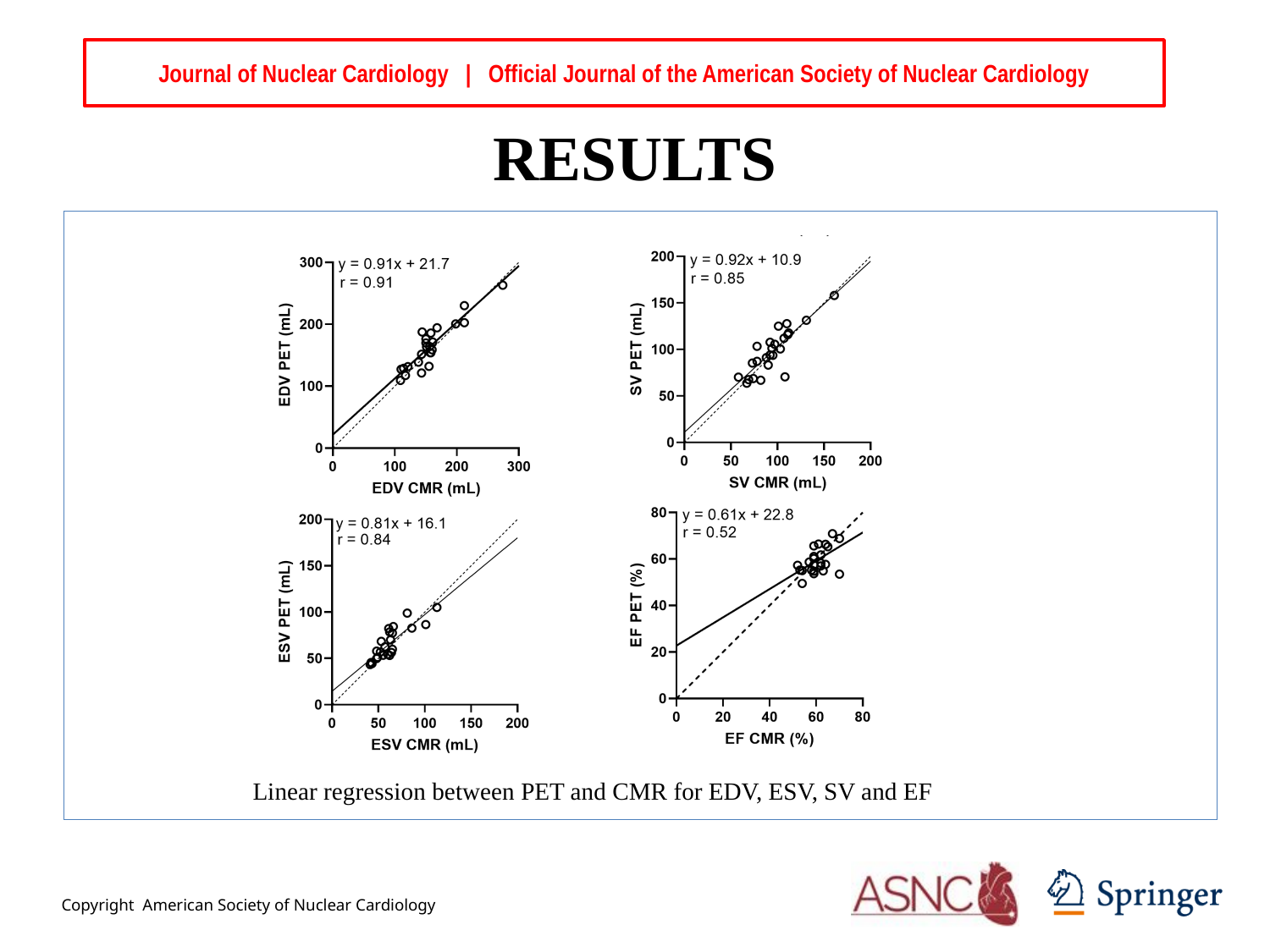

Journal of Nuclear Cardiology | Official Journal of the American Society of Nuclear Cardiology
# RESULTS
Linear regression between PET and CMR for EDV, ESV, SV and EF
Copyright American Society of Nuclear Cardiology

## Slide 5
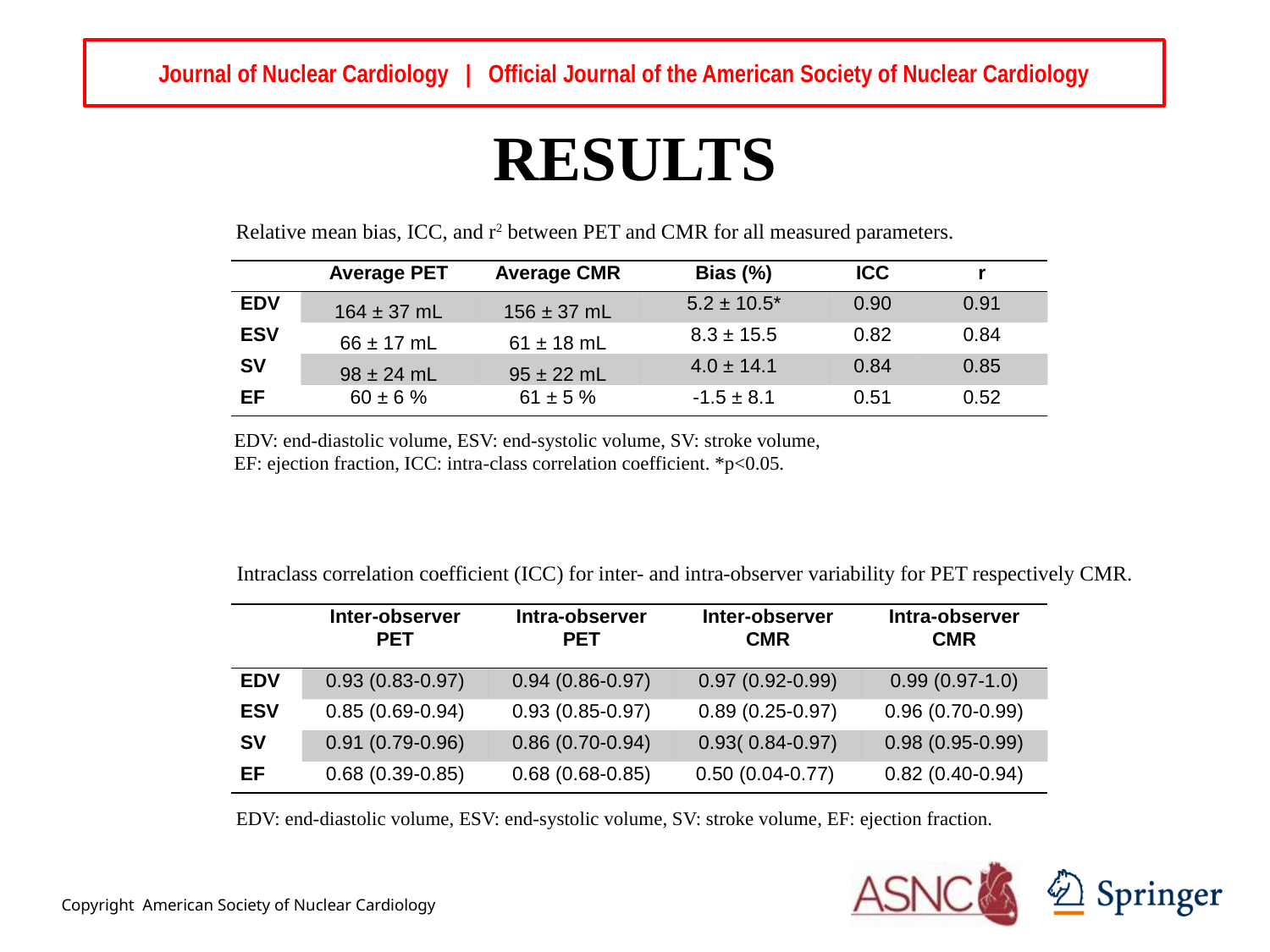

Journal of Nuclear Cardiology | Official Journal of the American Society of Nuclear Cardiology
# RESULTS
Relative mean bias, ICC, and r2 between PET and CMR for all measured parameters.
| | Average PET | Average CMR | Bias (%) | ICC | r |
| --- | --- | --- | --- | --- | --- |
| EDV | 164 ± 37 mL | 156 ± 37 mL | 5.2 ± 10.5\* | 0.90 | 0.91 |
| ESV | 66 ± 17 mL | 61 ± 18 mL | 8.3 ± 15.5 | 0.82 | 0.84 |
| SV | 98 ± 24 mL | 95 ± 22 mL | 4.0 ± 14.1 | 0.84 | 0.85 |
| EF | 60 ± 6 % | 61 ± 5 % | -1.5 ± 8.1 | 0.51 | 0.52 |
EDV: end-diastolic volume, ESV: end-systolic volume, SV: stroke volume,
EF: ejection fraction, ICC: intra-class correlation coefficient. *p<0.05.
Intraclass correlation coefficient (ICC) for inter- and intra-observer variability for PET respectively CMR.
| | Inter-observer PET | Intra-observer PET | Inter-observer CMR | Intra-observer CMR |
| --- | --- | --- | --- | --- |
| EDV | 0.93 (0.83-0.97) | 0.94 (0.86-0.97) | 0.97 (0.92-0.99) | 0.99 (0.97-1.0) |
| ESV | 0.85 (0.69-0.94) | 0.93 (0.85-0.97) | 0.89 (0.25-0.97) | 0.96 (0.70-0.99) |
| SV | 0.91 (0.79-0.96) | 0.86 (0.70-0.94) | 0.93( 0.84-0.97) | 0.98 (0.95-0.99) |
| EF | 0.68 (0.39-0.85) | 0.68 (0.68-0.85) | 0.50 (0.04-0.77) | 0.82 (0.40-0.94) |
EDV: end-diastolic volume, ESV: end-systolic volume, SV: stroke volume, EF: ejection fraction.
Copyright American Society of Nuclear Cardiology

## Slide 6
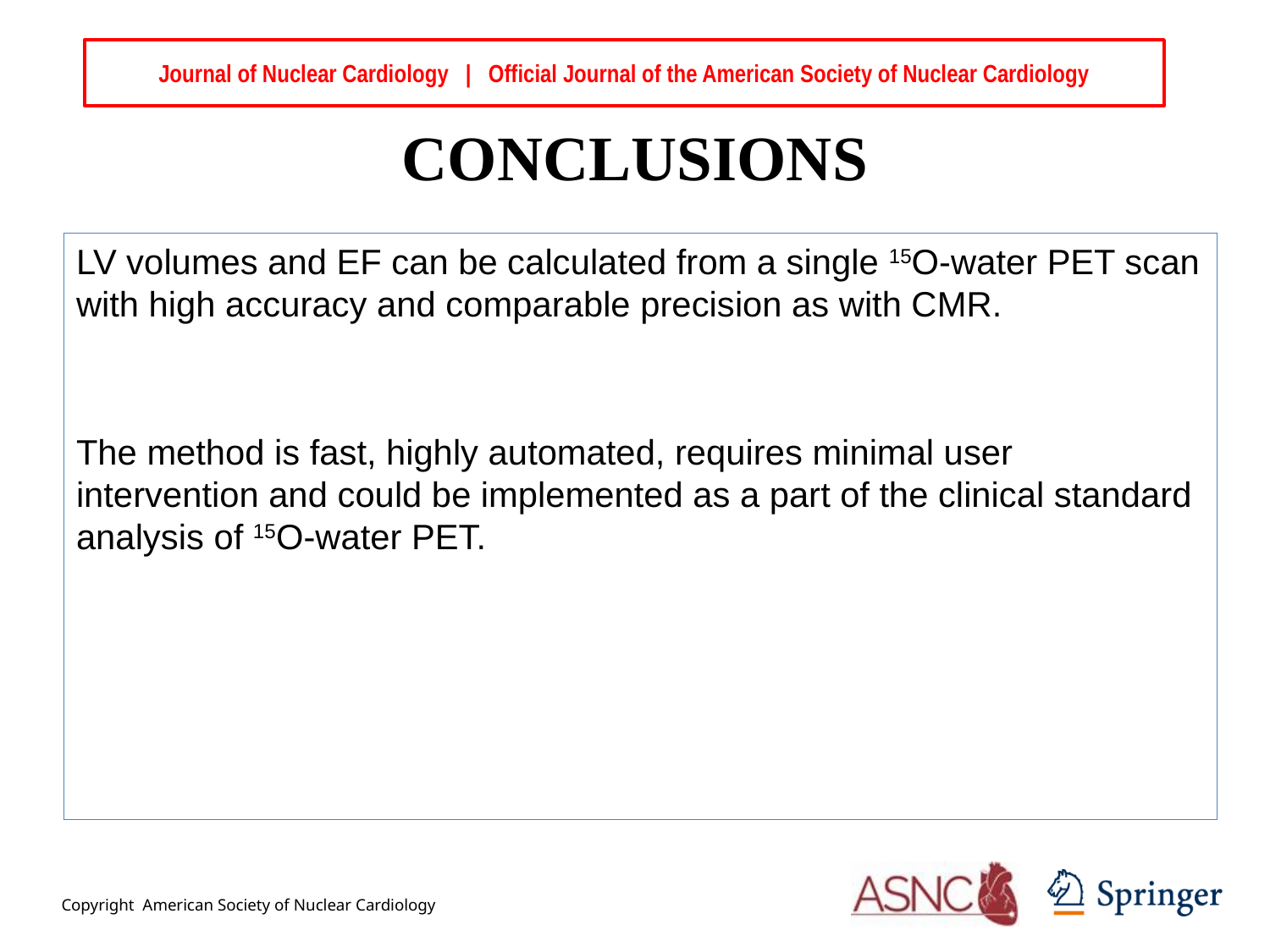

Journal of Nuclear Cardiology | Official Journal of the American Society of Nuclear Cardiology
# CONCLUSIONS
LV volumes and EF can be calculated from a single 15O-water PET scan with high accuracy and comparable precision as with CMR.
The method is fast, highly automated, requires minimal user intervention and could be implemented as a part of the clinical standard analysis of 15O-water PET.
Copyright American Society of Nuclear Cardiology
